# S-nitrosation on zinc finger motif of PARP-1 as a mechanism of DNA repair inhibition by arsenite

**DOI:** 10.18632/oncotarget.12613

**Published:** 2016-10-12

**Authors:** Xixi Zhou, Karen L. Cooper, Juliana Huestis, Huan Xu, Scott W. Burchiel, Laurie G. Hudson, Ke Jian Liu

**Affiliations:** ^1^ Department of Pharmaceutical Sciences, College of Pharmacy, University of New Mexico Health Sciences Center, Albuquerque, NM 87131, USA

**Keywords:** arsenic, DNA repair, PARP-1, zinc finger, reactive nitrogen species

## Abstract

Arsenic, a widely distributed carcinogen, is known to significantly amplify the impact of other carcinogens through inhibition of DNA repair. Our recent work suggests that reactive oxygen/nitrogen species (ROS/RNS) induced by arsenite (AsIII) play an important role in the inhibition of the DNA repair protein Poly(ADP-ribose) polymerase 1 (PARP-1). AsIII-induced ROS lead to oxidation of cysteine residues within the PARP-1 zinc finger DNA binding domain. However, the mechanism underlying RNS-mediated PARP inhibition by arsenic remains unknown. In this work, we demonstrate that AsIII treatment of normal human keratinocyte (HEKn) cells induced S-nitrosation on cysteine residues of PARP-1 protein, in a similar manner to a nitric oxide donor. S-nitrosation of PARP-1 could be reduced by 1400W (inducible nitric oxide synthase inhibitor) or c-PTIO (a nitric oxide scavenger). Furthermore, AsIII treatment of HEKn cells leads to zinc loss and inhibition of PARP-1 enzymatic activity. AsIII and 1400W/c-PTIO co-treatment demonstrate that these effects occur in an iNOS- and NO-dependent manner. Importantly, we confirmed S-nitrosation on the zinc finger DNA binding domain of PARP-1 protein. Taken together, AsIII induces S-nitrosation on PARP-1 zinc finger DNA binding domain by generating NO through iNOS activation, leading to zinc loss and inhibition of PARP-1 activity, thereby increasing retention of damaged DNA. These findings identify S-nitrosation as an important component of the molecular mechanism underlying AsIII inhibition of DNA repair, which may benefit the development of preventive and intervention strategies against AsIII co-carcinogenesis.

## INTRODUCTION

Arsenic is a co-carcinogen at low and non-cytotoxic concentrations. It inhibits DNA repair and greatly enhances the mutagenic, genotoxic and carcinogenic impact of other DNA-damaging agents, such as ultraviolet radiation (UVR) [1–7]. Certain zinc finger DNA repair proteins such as poly(ADP-ribose) polymerase-1 (PARP-1) are sensitive targets of trivalent arsenite (AsIII) [3, 5, 6, 8–12], and the resulting interactions between AsIII and these zinc finger proteins constitute an important mechanism underlying arsenic's inhibition of DNA repair. Particularly, cysteine residues on zinc finger motifs not only directly interact with AsIII [8], but also serve as redox-sensitive sites which are capable of altering protein structure and function as a result of oxidative modification by AsIII induced ROS [13, 14].

PARP-1 is a key DNA repair protein which plays an important role in multiple DNA repair pathways [15, 16]. The precise molecular mechanisms of PARP-1 inhibition by AsIII are still under investigation. It is known that AsIII selectively binds with C3H1 and C4 zinc fingers [8], and PARP-1 is a C3H1 zinc finger protein. Also, AsIII inhibits PARP-1 through reactive oxygen species (ROS) generation [13, 17, 18]. Importantly, our recent work suggests that these two mechanisms work together, leading to selective oxidation of C3H1 and C4 zinc finger proteins by AsIII binding [14]. In addition to ROS, AsIII exposure induces RNS production [4, 13, 19]. A previous study in HaCat cells shows that nitric oxide is produced with AsIII treatment [4]. AsIII-induced RNS contribute to PARP-1 inhibition as well as associated zinc loss [13], and blocking RNS production can rescue these effects of AsIII [13]. These findings suggest that apart from selective oxidation by AsIII derived from ROS production and zinc finger binding, there is an important RNS-mediated mechanism that needs to be defined. In particular, it is of great interest to know whether RNS directly target PARP-1 protein, and, if so, the manner in which this interaction of RNS with PARP-1 takes place. These questions are central to understanding the precise molecular mechanism of AsIII inhibition of PARP-1 through RNS production.

In this study, we present evidence that AsIII-generated RNS induces S-nitrosation on PARP-1. S-nitrosation describes the reaction in which cysteine residues are converted into S-nitroso-cysteine on a thiol group. This is a post-translational modification that regulates protein functions and is involved in various cell signaling mechanisms of RNS [20, 21]. We found that AsIII-induced RNS caused S-nitrosation of cysteine residues on the zinc finger DNA binding domain of PARP-1, resulting in loss of zinc and protein function. This observation provides a mechanistic basis for an RNS-dependent pathway of PARP-1 inhibition by AsIII, which adds important insight into the molecular mechanisms underlying arsenic-mediated DNA repair inhibition.

## RESULTS

### Reactive nitrogen species (RNS) contribute to AsIII enhancement of UV-induced DNA damage

**Figure 1:**
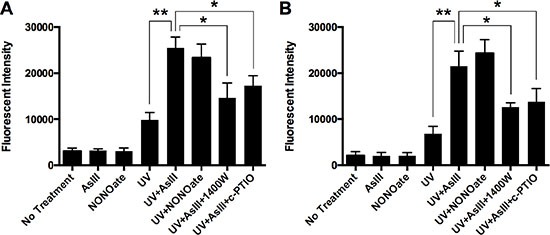
RNS contribute to AsIII enhanced UV-induced DNA double-strand break (**A**) HEKn cells were treated by 10 μM NONOate, 1 μM sodium AsIII, 100 μM 1400W with 1 μM AsIII, or 100 μM carboxy-PTIO (c-PTIO) with 1 μM AsIII for 24 h. In some samples, cells were exposed to 2 kJ/m2 UV radiation at 4 hrs before collection. Phospho-H2AX level was then measured by flow cytometry, represented by Mean Channel Fluorescence (MCF). AsIII significantly increased UV-induced DNA double strand break, which could be reduced by iNOS inhibition (1400 W) or NO scavenging (c-PTIO) (**B**) HaCat cells were treated with the same conditions. Similar profiles of phospho-H2AX levels were observed. Barcharts show MCF ± SD; *p < 0.05, **p < 0.01 (student's *t*-test); *n* = 3.

Arsenic amplifies DNA damage caused by UV-radiation [1, 22]. It is also reported that arsenic generates RNS [4, 13]. To demonstrate the contribution of RNS to AsIII enhancement of UV-induced DNA damage, we quantified DNA double strand break (DSB) in the presence of iNOS inhibitor or NO scavenger. HEKn cells were treated with 10 μM NONOate (nitric oxide donor), 1 μM AsIII, 1 μM AsIII with 100 μM 1400 W, or 1 μM AsIII with 100 μM c-PTIO (carboxy-PTIO, nitric oxide scavenger) for 24 h. A subset of cells were exposed to 2 kJ/m2 solar-simulated UV radiation. Flow cytometry analysis of phospho-H2AX (a marker of DSB, also known as gamma-H2AX [23]) was performed 4 h after UV treatment. Figure 1A shows that AsIII significantly amplified UV-induced DSB in HEKn cells. However, iNOS inhibition by 1400 W partially reversed this effect, which showed the contribution of iNOS. NO scavenging by c-PTIO also partially reversed the effect of AsIII, indicating that iNOS contribution is likely to be through NO generation. To confirm the findings, we performed similar experiments in the spontaneously immortalized keratinocyte line (HaCat). Although HaCat cells are widely used as a substitution for normal keratinocytes, there are reports showing that HaCat cells behave differently than normal keratinocytes in some respects, such as response to UV [24] or oxidative stress [25]. In HaCat cells, we observed similar results as in HEKn cells (Figure 1B). These results showed the contribution of RNS to AsIII enhancement of UV-induced DNA damage and are consistent with our previous report [4].

### RNS as a mechanism of PARP-1 inhibition by AsIII

**Figure 2:**
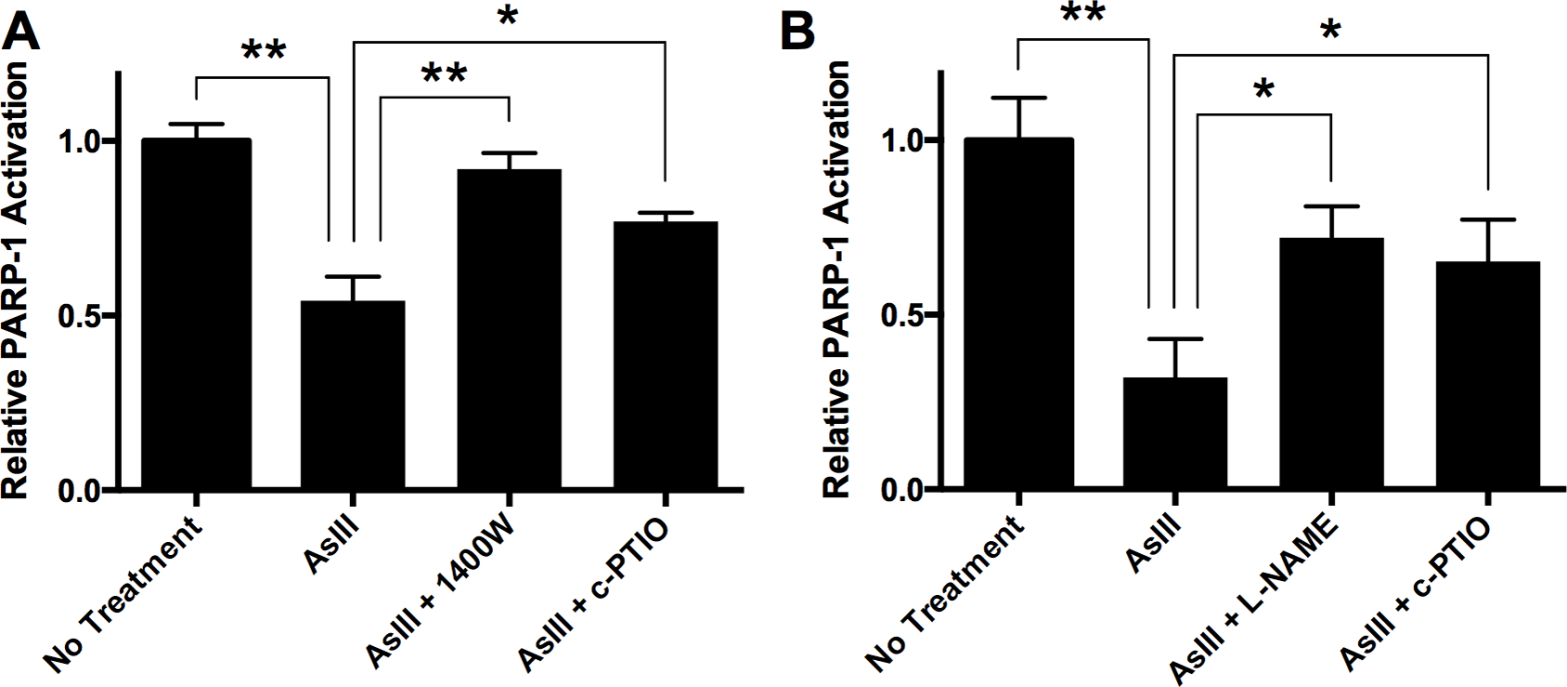
AsIII inhibited PARP-1 activity in an RNS-dependent manner (**A**) In HEKn cells, iNOS induction and NO production contributes to PARP-1 activity inhibition by AsIII. HEKn cells were treated with 1 μM sodium AsIII, 100 μM 1400W with 1 μM AsIII, or 100 μM carboxy-PTIO (c-PTIO) with 1 μM AsIII for 24 h. Then PARP-1 activity was measured as described in the methods section. AsIII inhibited PARP-1 activity, but inhibition of iNOS (1400W) or scavenging of NO (c-PTIO) rescued AsIII-inhibited PARP-1 activity. (**B**) Similar results are shown for HaCat cells. HaCat cells were treated by 1 μM sodium AsIII, 100 μM L-NAME with 1 μM AsIII, or 100 μM carboxy-PTIO (c-PTIO) with 1 μM AsIII for 24 h. NOS inhibition by L-NAME and NO scavenging by c-PTIO rescued AsIII-inhibited PARP-1 activity. Barcharts show mean ± SD; *p < 0.05, **p < 0.01 (student's *t*-test); *n* = 3.

It is reported that PARP-1 inhibition is responsible for the amplification of UV-induced DNA damage by AsIII, and PARP-1 is a sensitive target of AsIII [6, 8, 11]. To demonstrate that RNS play an important role in PARP-1 inhibition by AsIII, we tested the effect of an iNOS inhibitor and NO scavenger on PARP-1 activity inhibition by AsIII. HEKn cells were treated with AsIII, AsIII with 1400W, or AsIII with c-PTIO for 24 h. Then PARP-1 activity was measured using the HT colorimetric PARP-1 activity assay (Figure 2A). 1 μM AsIII at 24 h significantly inhibited PARP-1 activity, which is consistent with our previous work [5]. iNOS inhibition by 1400W or NO scavenging by c-PTIO significantly rescued PARP-1 activity (Figure 2A), which indicates that iNOS-produced NO plays an important role in AsIII inhibition of PARP-1 activity. Similar results were found in HaCat cells (Figure 2B). These results indicate that AsIII inhibited PARP-1 activity, and NOS inhibition or NO scavenging partially rescued PARP-1 activity inhibited by AsIII, demonstrating the importance of a RNS-mediated mechanism in AsIII inhibition of PARP-1.

### AsIII induces S-nitrosation of PARP-1 through iNOS induction

**Figure 3:**
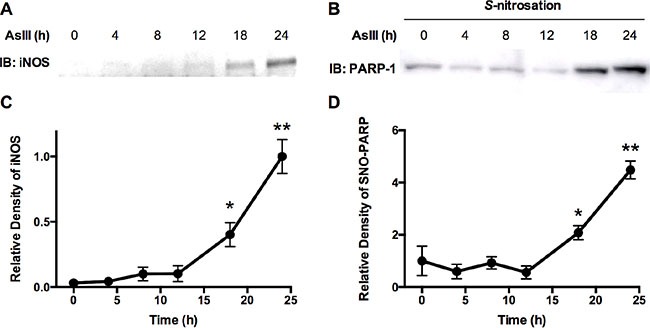
AsIII-induced S-nitrosation on PARP-1 protein correlates to iNOS expression in cells (**A**) Immunoblotting analysis of iNOS expression time course. HEKn cells were treated with 1 μM AsIII. iNOS protein levels were then analyzed by immunoblotting at indicated time points. (**B**) PARP-1 S-nitrosation time course. HEKn cells were treated with 1 μM AsIII. At the indicated time points, cells were lysed and the modified biotin-switch method was applied to purify S-nitrosated proteins, from which S-nitrosated PARP-1 was then analyzed by immunoblotting. (**C**) and (**D**) are densitometric analyses of immunoblotting results (A) and (B), respectively, using β-actin and total PARP-1 protein as corresponding loading controls. iNOS was significantly induced starting from 18 hrs. At 24 h, iNOS protein level was about 30 times higher than 0 h. PARP-1 S-nitrosation also significantly increased, starting from 18 h of AsIII treatment. At 24 h, S-nitrosation of PARP-1 increased more than 4 fold compared to 0 h. Plots show mean ± SD; *p < 0.05, **p < 0.01 (student's *t*-test vs. 0 h); *n* = 3.

We reported previously that AsIII induces iNOS expression and NO production at 24 h in HaCat cells [4]. Here in normal human keratinocytes (HEKn), we tested the time-course of iNOS induction in order to confirm that NO signaling can be induced by AsIII on the level of iNOS protein expression, as well as to establish the time for onset of induction. HEKn cells were treated with 1 μM AsIII, then iNOS expression was analyzed by immunoblotting at multiple time points (Figure 3A). From 0 h to 12 h, iNOS was not significantly induced by AsIII. The earliest time point of significant iNOS induction was 18 h, with nearly a 10-fold induction compared to untreated controls (Figure 3C). At 24 h, iNOS was further induced achieving nearly 30-fold induction. Since NO production is mainly regulated by protein expression of iNOS [26], this shows that AsIII may induce NO signaling by up-regulating iNOS at the protein level in HEKn cells, which is consistent with our previous study in HaCat cells [4].

It is known that S-nitrosative modification on cysteine is an important mechanism in RNS function [20], so we examined whether AsIII induces S-nitrosation on PARP-1. Furthermore, to determine the time point of significant S-nitrosation induction by AsIII, and demonstrate the correlation between S-nitrosation and iNOS induction, we performed a time course analysis of PARP-1 S-nitrosation by AsIII. HEKn cells were treated with 1 μM AsIII. At multiple time points, cells were lysed and S-nitrosated proteins were purified by a modified biotin-switch assay as described in the methods section. Immunoblotting using anti-PARP-1 antibody on purified S-nitrosated protein indicated S-nitrosation levels on PARP-1 protein (Figure 3B). Densitometric analysis showed that PARP-1 S-nitrosation was induced by approximately 2-fold starting from 18 h of AsIII treatment (Figure 3D), when compared to control (0 h). At 24 h of AsIII treatment, S-nitrosation on PARP-1 protein was increased by more than 4-fold (Figure 3D). This result not only showed that AsIII induces S-nitrosative modification on PARP-1 protein, but also demonstrated that S-nitrosation of PARP-1 corresponded well with iNOS induction in time scale (Figure 3C and 3D).

### AsIII induces S-nitrosation of PARP-1 in an RNS-dependent manner

**Figure 4:**
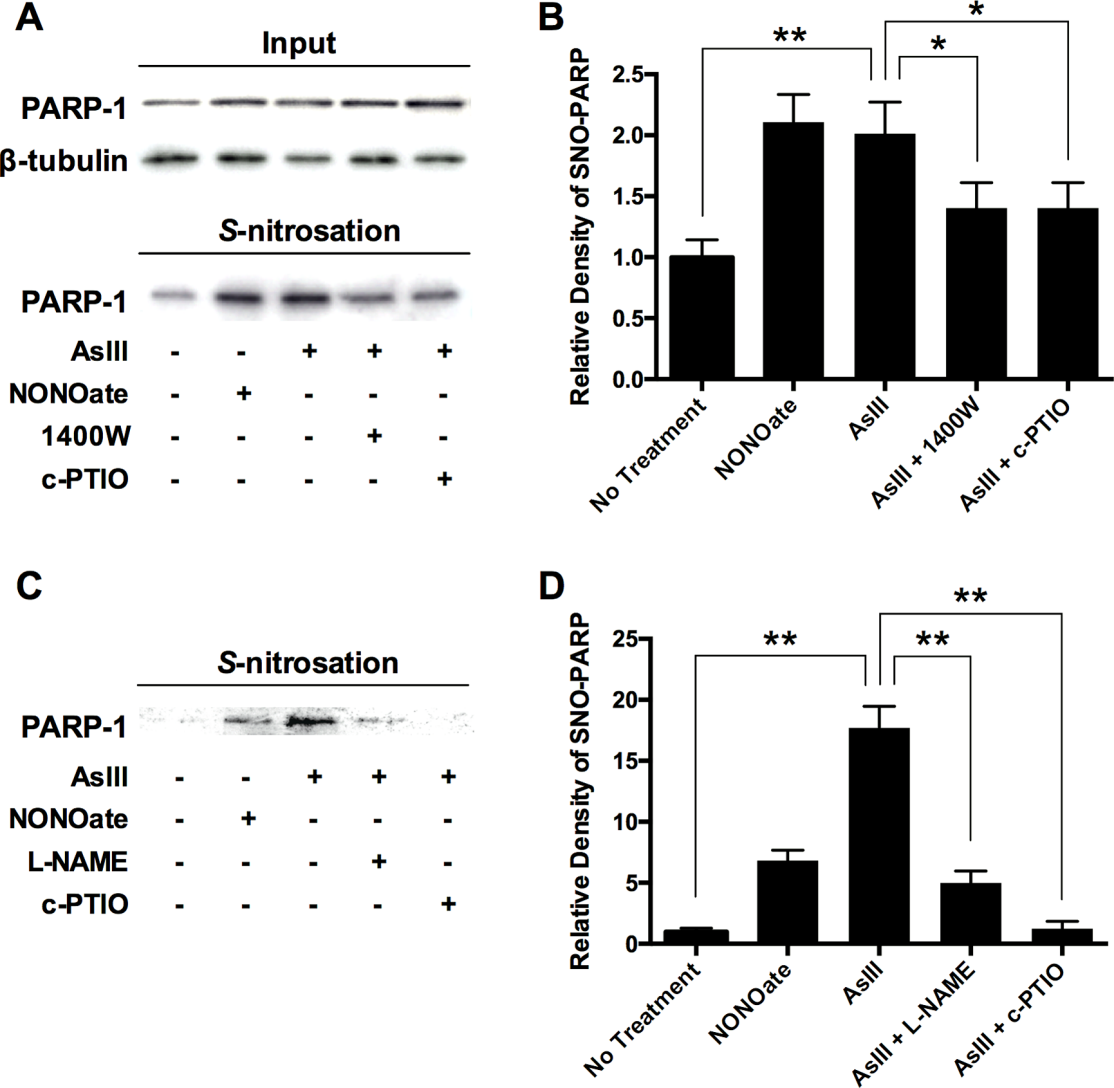
AsIII-induced PARP-1 S-nitrosation resulting from RNS induction (**A**) AsIII-induced PARP-1 S-nitrosation in an iNOS- and NO-dependent manner. HEKn cells were treated with 10 μM NONOate (NO donor), 1 μM sodium AsIII, 100 μM 1400W (iNOS inhibitor) with 1 μM AsIII, or 100 μM carboxy-PTIO (c-PTIO, NO scavenger) with 1 μM AsIII for 24 h. The various treatments did not change PARP-1 protein level (top panels). PARP-1 S-nitrosation was analyzed by modified biotin-switch assay and immunoblotting. (**B**) Densitometric analysis of bottom panel (A). AsIII induced PARP-1 S-nitrosation, as did NONOate. Inhibiting iNOS or scavenging NO decreased AsIII-induced PARP-1 S-nitrosation. (**C**) Similar results are obtained in HaCat cells. AsIII-induced S-nitrosation on PARP-1 was significantly blocked by NOS inhibition (L-NAME) or NO scavenging (NONOate). (**D**) Densitometric analysis of bottom panel (C). Barcharts show mean ± SD; *p < 0.05, **p < 0.01 (student's *t*-test); *n* = 3.

The time course correlation between iNOS induction and PARP-1 S-nitrosation indicates that S-nitrosation on PARP-1 may be the result of RNS induced by AsIII. To further confirm this mechanism, we used the iNOS inhibitor 1400W and the NO scavenger carboxy-PTIO (c-PTIO) to co-treat with AsIII in HEKn cells. HEKn cells were treated with NONOate, AsIII, AsIII with 1400 W, or AsIII with c-PTIO for 24 h. Total PARP-1 protein level was consistent across all conditions (Figure 4A, top panel). S-nitrosated PARP-1 was then analyzed using a modified biotin-switch assay paired with immunoblotting (Figure 4A, bottom panel). Densitometric analysis showed that AsIII significantly induced PARP-1 S-nitrosation, equivalent to the effect of NONOate (Figure 4B). However, inhibiting iNOS or scavenging NO significantly reduced PARP-1 S-nitrosation by AsIII (Figure 4B), thus clearly indicating that AsIII-induced S-nitrosation of PARP-1 occurred through iNOS activation and NO production. Similar results were obtained in HaCat cells (Figure 4C, 4D), indicating that S-nitrosation of PARP-1 is the result of AsIII-induced RNS.

### AsIII induces S-nitrosation on PARP-1 zinc finger DNA-binding domain

**Figure 5:**
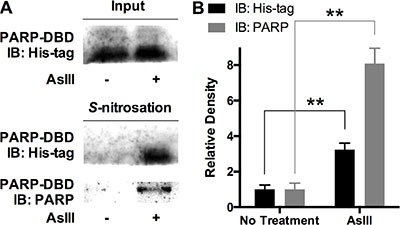
AsIII induced S-nitrosation of PARP-1 on zinc finger DNA binding domain (DBD) His-tagged PARP-1-DBD was expressed in HEKn cells for 48 h. Then cells were treated with 2 μM AsIII for 24 h. (**A**) PARP-1-DBD expression was not altered by AsIII treatment (top panel). Immunoblotting on modified biotin-switch assay purified S-nitrosated proteome showed that AsIII induced S-nitrosation on PARP-1-DBD. Middle panel, immunoblotting with anti-His-tag antibody; bottom panel, immunoblotting with anti-PARP antibody. (**B**) Densitometric analysis of S-nitrosation blottings. Barchart shows mean ± SD; **p < 0.01 (student's *t*-test); *n* = 3.

The results from the studies described above indicate that PARP-1 S-nitrosation induced by iNOS-produced NO may be a key event in PARP-1 inhibition of AsIII. PARP-1 is a zinc finger DNA repair protein. The cysteine-rich zinc finger motif serves as a key structure in DNA binding, which is critically important to PARP-1 DNA recognition and DNA repair activity [27, 28]. Zinc binding to the PARP-1 zinc finger motif is essential for the maintenance of three-dimensional protein structure [29], while zinc loss is an important component of PARP-1 inhibition by AsIII [9]. From a chemical perspective, S-nitrosation on PARP-1 should occupy cysteine residues so that zinc may no longer be able to bind to PARP-1 protein. Nevertheless, in PARP-1 protein, there are two free cysteine residues located outside of the zinc finger DNA-binding domain (DBD); other cysteine residues all bind with zinc and are located within the DBD [29]. In order to demonstrate the direct link between S-nitrosation and zinc loss, we must present evidence that AsIII induced S-nitrosation on PARP-1-DBD. Therefore, we analyzed S-nitrosation by AsIII on PARP-1 zinc finger DNA-binding domain (DBD). We over-expressed His-tagged PARP-1-DBD in HEKn cells. His-tagged PARP-1-DBD was expressed in HEKn cells for 48 h. The cells were then treated with 1 μM AsIII for 24 h. AsIII treatment did not alter the expression level of PARP-1-DBD (Figure 5A, top panel). However, S-nitrosation of PARP-1-DBD analyzed by biotin-switch assay showed that PARP-1-DBD was S-nitrosated by AsIII treatment (Figure 5A bottom panel). Both anti-His-tag and anti-PARP antibodies confirmed the significant elevation of S-nitrosation on PARP-1-DBD (densitometric results and statistics are shown in Figure 5B). This result shows that zinc-binding cysteine residues on PARP-1 are targets of RNS-induced S-nitrosation by AsIII.

### AsIII induces zinc loss from PARP-1 protein in an RNS-dependent manner

**Figure 6:**
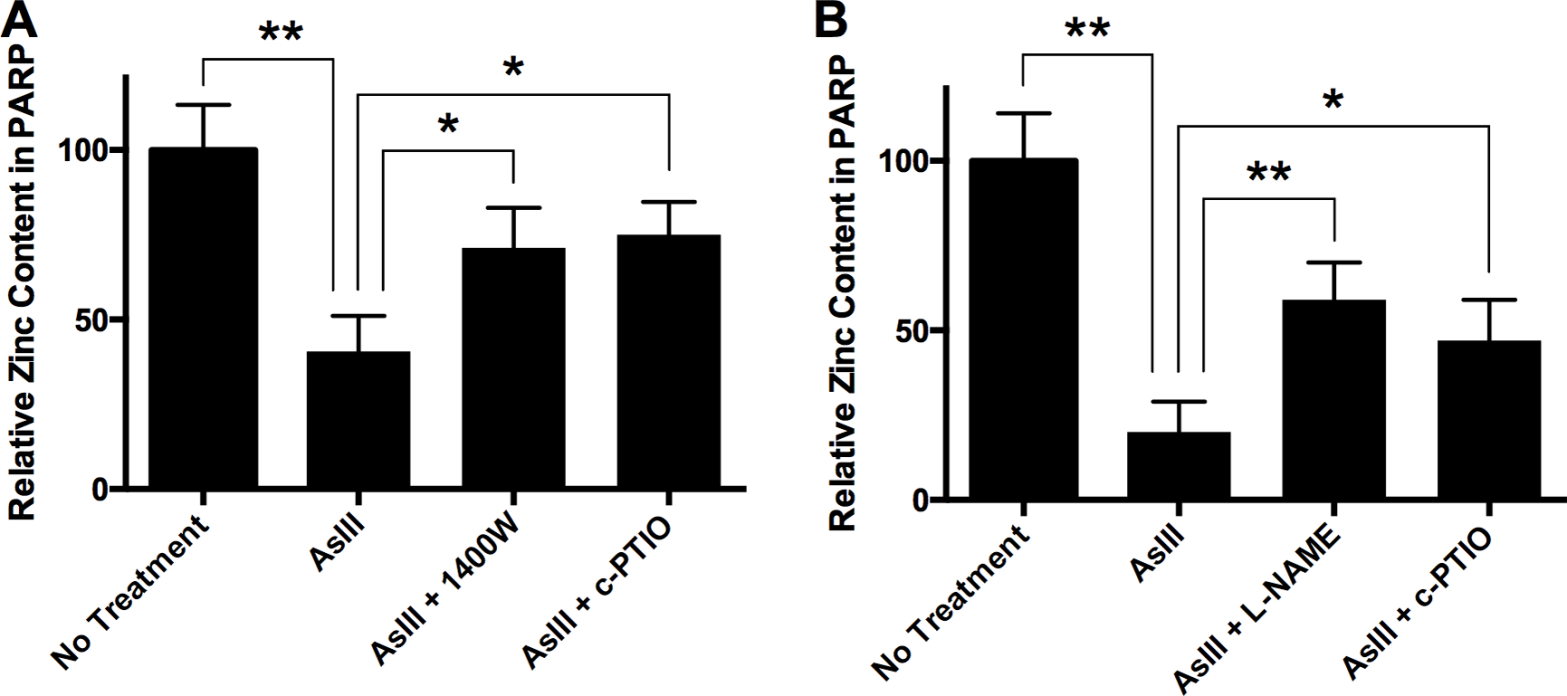
AsIII induces zinc loss from PARP-1 through RNS (**A**) In HEKn cells, iNOS and NO contributed to AsIII-induced zinc loss from PARP-1. HEKn cells were treated with 1 μM sodium AsIII, 100 μM 1400W with 1 μM AsIII, or 100 μM carboxy-PTIO (c-PTIO) with 1 μM AsIII for 24 h. Zinc content in PARP-1 was then measured as described in the methods section. AsIII caused zinc loss from PARP-1. However, inhibition of iNOS (1400W) or scavenging of NO (c-PTIO) rescued zinc content in PARP-1. (**B**) Similar results were seen in HaCat cells. HaCat cells were treated with 1 μM sodium AsIII, 100 μM L-NAME with 1 μM AsIII, or 100 μM carboxy-PTIO (c-PTIO) with 1 μM AsIII for 24 h. NOS inhibition by L-NAME and NO scavenging by c-PTIO attenuated AsIII-caused zinc loss from PARP-1. Barcharts show mean ± SD; *p < 0.05, **p < 0.01 (student's *t*-test); *n* = 3.

To further confirm that AsIII-induced zinc loss from PARP-1 protein is RNS-dependent, we analyzed zinc content in PARP-1 protein. HEKn cells were treated by 1 μM AsIII, 1 μM AsIII with 100 μM 1400W, or 1 μM AsIII with 100 μM c-PTIO for 24 h. Then cells were collected and PARP-1 protein was purified by immunoprecipitation. Zinc content was measured by 4-(2-pyridylazo)resorcinol (PAR) assay [30, 31]. AsIII caused dramatic zinc loss from PARP-1 protein (Figure 6A). Adding iNOS inhibitor or NO scavenger to AsIII treatment significantly restored zinc content in PARP-1 protein. A similar result was observed in HaCat cells: AsIII-induced RNS contributed to zinc loss from PARP-1 protein. This result, together with the previous PARP-1 activity analysis (Figure 2), suggested that S-nitrosation on the zinc finger motif is a mechanism of AsIII induced zinc loss from PARP-1, which provides a possible explanation for AsIII-induced loss of PARP-1 function.

## DISCUSSION

**Figure 7:**
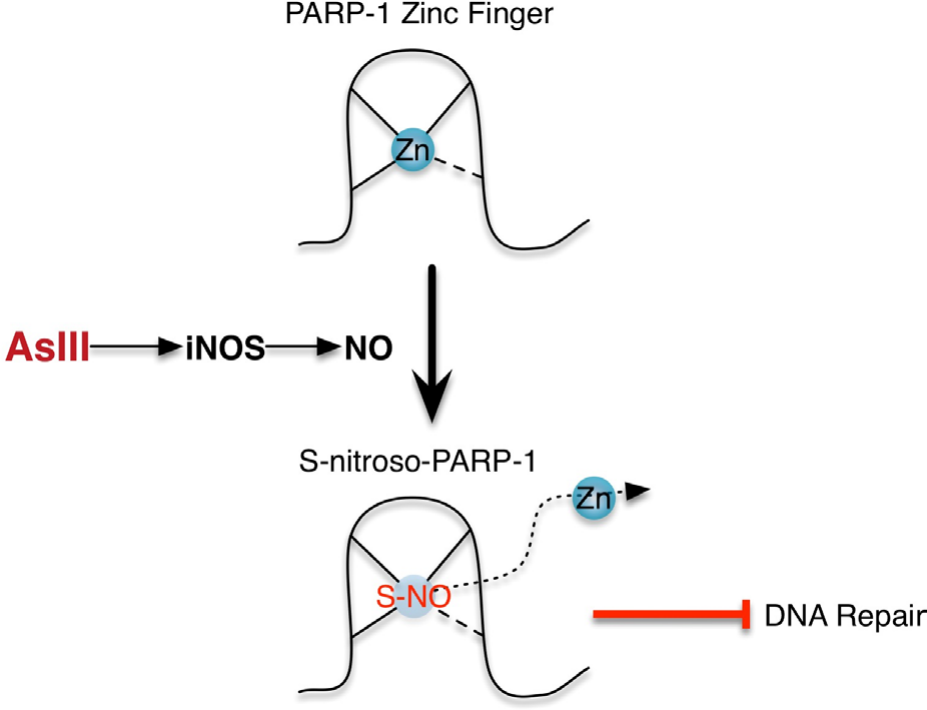
Schematic illustration of the NO mechanism of PARP-1 inhibition by AsIII AsIII exposure induces iNOS, which produces NO. NO causes S-nitrosation of PARP-1, especially on cysteine residues located within the PARP-1 zinc finger DNA binding domain, leading to zinc loss and dysfunction of PARP-1.

This work demonstrates a clear mechanism for the participation of RNS in AsIII inhibition of DNA repair protein PARP-1. As schematically illustrated in Figure 7, AsIII induces iNOS, which produces NO, resulting in S-nitrosation on PARP-1 zinc finger, which contributes to zinc loss and protein dysfunction. S-nitrosation of PARP-1 is a key event in this mechanism, which provides a connection between two known aspects of AsIII-mediated co-carcinogenesis: the generation of RNS, and the induction of PARP-1 zinc loss and dysfunction.

In the present study we have demonstrated that AsIII amplifies DNA damage, and inhibits PARP-1 activity in an RNS-dependent manner (Figures 1 and 2). It is reported that AsIII induces iNOS in HaCat cells at 24 h, and that NO has been generated in this context [4]. All of these results suggest that RNS play a role in DNA repair inhibition, and interact with PARP-1 protein. Here for the first time, we demonstrate that AsIII-induced RNS interact with PARP-1 through S-nitrosation of zinc finger cysteines. First, both AsIII and the NO donor NONOate were capable of S-nitrosating PARP-1, (Figure 4), showing that AsIII is equivalent to the NO donor in terms of modifying PARP-1. Second, S-nitrosation of PARP-1 synchronized with iNOS induction after AsIII treatments (Figure 3C and 3D). The time course experiments showed that both iNOS protein level and PARP-1 S-nitrosation significantly increased starting from 18 h, and further increased at 24 h. This result clearly indicates that S-nitrosation of PARP-1 is related to iNOS induction by AsIII. A decrease in PARP-1 S-nitrosation results from iNOS inhibition or NO scavenging (Figure 4B), which indicates that S-nitrosation of PARP-1 by AsIII is caused by iNOS induction and NO production. Taken together, these results demonstrate that S-nitrosation is a specific protein modification to PARP-1 resulting from AsIII-induced RNS.

Furthermore, we demonstrated that S-nitrosation of PARP-1 is responsible for the RNS mechanism of AsIII-induced zinc loss, thus leading to loss of PARP-1 function. We showed that AsIII treatment caused S-nitrosation on the PARP-1 zinc finger DNA binding domain, which indicated that S-nitrosation is the mechanism of RNS-induced zinc loss from PARP-1, resulting in inhibition of PARP-1 activity. In PARP-1 protein, zinc finger domains associate with zinc ions in order to maintain protein conformation, and function as a critical structure for DNA recognition and binding. Binding of each zinc ion requires one histidine and three cysteine residues, and Zn interacts with the Cys residues by forming a S-Zn coordination bond. When S-NO forms, the S-Zn coordination bond is disrupted, demonstrating that Zn is not capable of binding with S-nitrosated Cys. All cysteine residues within PARP-1 -DBD are located in a zinc finger motif and are necessary for Zn binding, leaving no free cysteine residues present in the PARP-1-DBD when it is fully occupied with Zn [29]. Therefore, AsIII-induced S-nitrosation on the PARP-1-DBD directly abolishes zinc binding capability, which provides an explanation for zinc loss and PARP-1 inhibition secondary to RNS production. PARP-1 has other free cysteine residues located on regulatory domain, which may also be S-nitrosated. However, our previous report showed that PARP-1 DNA binding activity was inhibited by NO [13]. Here we found that zinc content in PARP-1 protein was reduced by AsIII in an NO- and iNOS-dependent manner. Modifications on cysteine residues in zinc finger DNA binding domains are directly related to PARP-1 DNA binding activity and zinc content. Thus, we believe S-nitrsosation on zinc finger DNA binding domain to be more important as a molecular mechanism of PARP-1 inhibition by AsIII. We observed that AsIII-induced zinc loss from PARP-1 protein was rescued by iNOS inhibition or NO scavenging (Figure 6). These results suggest that S-nitrosation is a key event in the AsIII-induced RNS mechanism of PARP-1 inhibition and zinc loss. In addition, for PARP-1 activity inhibition, S-nitrosation and zinc loss by AsIII, similar results were observed in both HEKn cells and HaCat cells, which indicated that the RNS mechanism is not cell-line specific in keratinocytes.

Our previous work showed that ROS induction and AsIII binding with the PARP-1 zinc finger work together to inhibit PARP-1 [14]. The nitrosative mechanism described in this paper should not be interpreted as a replacement for the previously described oxidative mechanism. Instead, the role of S-nitrosation is one further piece of the whole picture of PARP-1 inhibition by AsIII. In this work, we saw that iNOS inhibition and NO scavenging only partially rescued the effect of AsIII on PARP-1 activity and zinc content, indicating that nitrosative modifications are not solely responsible for these effects and, similarly to the ROS story [14], may synergize with other mechanisms as well. Furthermore, AsIII and an NO donor had similar effects on PARP-1 activity inhibition (Figure 2A). However, the NO donor was not as effective as AsIII in causing zinc loss from PARP-1 (Figure 6A). This result suggests that RNS may work with other related mechanisms to inhibit PARP-1. The relationship between RNS-mediated changes and other mechanisms underlying AsIII-related changes to PARP-1 structure and activity is of interest, and will be important to investigate in future studies. Another interesting observation comes from the time course for AsIII-induced RNS and S-nitrosation. Herein we saw that iNOS induction and S-nitrosation were significantly elevated from 18 h of AsIII treatment onward. In contrast, ROS production by AsIII was reported to be induced within 2 h [14]. The different tempos of ROS/RNS induction by AsIII, and their potential synergy and relative contribution to the chronic effect of AsIII inhibition of DNA repair are important to explore in future studies. S-nitrosation itself is a complex mechanism. A previous report showed that AsIII inhibits iNOS and overall S-nitrosation under a different condition [32]. Thus, the particular sensitivity of PARP-1 to NO and S-nitrosation is to be further investigated. Therefore, PARP-1 S-nitrosation by AsIII represents a critical component of AsIII inhibition of PARP-1 through RNS, and more importantly, should greatly benefit our understanding of the overall molecular mechanism of DNA repair inhibition by arsenic. These findings indicate that targeting NO and protein S-nitrosation could be an effective approach to limiting the carcinogenic activity of As exposure. For example, using an NO scavenger or NOS inhibitor may attenuate DNA damage, PARP-1 activity inhibition and zinc loss caused by AsIII.

The S-nitrosation of PARP-1 on cysteine residues within zinc finger motifs underscores the importance of cysteine residues in relation to AsIII toxicity. These residues are naturally occupied by zinc ions, ensuring the correct structure and normal function of PARP-1, but in the context of AsIII exposure are subject both to oxidative/nitrosative modifications and to direct interaction with AsIII. The disruption of cysteine residues results in loss of PARP-1 function. Therefore, research into the relationship between different pathways of PARP-1 inhibition by AsIII must ultimately investigate AsIII-mediated cysteine modifications. The identification of S-nitrosation is an important step in this direction, which may ultimately include a survey of oxidation forms, as well as investigations into the conversions between oxidation/nitrosation forms and the reversibility of these modifications. This line of inquiry will contribute greatly to the study of mechanisms underlying DNA repair inhibition by AsIII.

In summary, PARP-1 S-nitrosation by AsIII constitutes an important molecular mechanism by which AsIII inhibits PARP-1 through RNS. The findings may benefit not only our understanding of the mechanism of AsIII inhibition of DNA repair, but also future investigations into prevention of and intervention into AsIII co-carcinogenesis.

## MATERIALS AND METHODS

### Cell culture and chemicals

Normal human neonatal epidermal keratinocytes (HEKn) and DermaLife K culture medium were purchased from Lifeline Cell Technologies (Oceanside, CA). HEKn cells were grown and maintained according to manufacturer's instructions. The human keratinocyte cell line (HaCat) was a kind gift from Dr. Mitch Denning (Loyola University Medical Center, Maywood, IL). HaCat cells were maintained as described previously [5]. Sodium arsenite (> 99%) was obtained from Fluka Chemie. NONOate, 1400W, L-NAME and carboxy-PTIO (c-PTIO) were obtained from Cayman Chemical. Other chemicals were obtained from Sigma-Aldrich unless otherwise indicated.

### UV radiation and flow cytometry assay for phospho-H2AX

HEKn cells or HaCat cells were treated as indicated for 20 h, then were exposed to 2 kJ/m2 solar-simulated UV radiation (ss-UVR) using an Oriel 300 W Solar Ultraviolet Simulator (Newport Corporation, Irvine, CA). After exposure to ss-UVR, cells were returned to the incubator for an additional 4 h, then harvested and washed with ice-cold phosphate-buffered saline (PBS). To fix the cells, a freshly made solution of 4% formaldehyde was added to the suspension cells, which were then incubated for 10 min at room temperature. Cells were permeabilized with −20°C 90% methanol for 20 min at room temperature after another wash with PBS. Then, cells were washed again and stained with 50 mL per sample of antibody dilution at room temperature for 1 h in the dark. Alexa Fluor 647 mouse anti-H2AX (pS139) was purchased from BD Biosciences (San Jose, CA). Antibody was made into a 1:10 dilution with PBS immediately before use. Stained cells were washed, rinsed and resuspended with PBS and analyzed using a BD Accuri C6 flow cytometer. At least 20,000 cells have been analyzed in each sample for fluorescent intensity analysis. Control antibody: APC Mouse IgG1 κ Isotype Control (BD Biosciences, San Jose, CA).

### PARP activity assay

HEKn cells were treated as described in figure legends and the results section. Cells were then collected using RIPA buffer and the protein was extracted. Protein extraction procedures were identical to the corresponding steps in the biotin-switch assay, and the concentrations were determined with the BCA protein detection kit (Thermo Fisher Pierce). PARP activity was measured using the HT Colorimetric PARP/Apoptosis Assay kit (Trevigen, Gaithersburg, MD). The experiments were performed following the manufacturer's instructions. The relative activity value was presented using the absorbance at 450 nm on a SpectraMax 450 plate reader.

### Immunoblotting assay

Samples were resolved by electrophoresis through 10% SDS-polyacrylamide gels. Proteins were transferred to nitrocellulose membranes (Bio-Rad) and probed with corresponding antibodies. Antibodies used in this work are as follows: iNOS (Santa Cruz Biotechnology, SC-7271), PARP-1 (Cell Signaling Inc. #9532), β-tubulin (Santa Cruz Biotechnology, SC-55529), and His-tag (Cell Signaling Inc. #2365). The membranes were developed using the SuperSignal chemiluminescent detection system (Thermo Scientific Pierce). Quantification of immunoblotting results was performed using software ImageJ. Statistical summaries were obtained from at least 3 independent samples.

### Analysis of protein S-nitrosation with modified biotin-switch assay

This assay is modified from the “biotin-switch” assay [33–35]. All following steps were performed in the dark. Treated HEKn cells were harvested in RIPA cell lysis buffer (Thermo Scientific), sonicated, and centrifuged at 13,500 rpm for 15 min at 4°C to remove cellular debris. 10 mM N-ethylmaleimide (NEM; Sigma-Aldrich) was added and incubated for 1 h at room temperature to block all free Cys residues. Excess NEM in samples was removed by ice-cold acetone precipitation repeated three times for 20 min each. 1 mM ascorbic acid (Vc, Sigma-Aldrich) and 500 μM biotin-NEM (Thermo Scientific Pierce) were then added and incubated at room temperature for 1 h in order to reduce S-nitrosated Cys on proteins and label the residues. Labeled S-nitrosated proteome was purified using streptavidin agarose beads (Thermo Scientific Pierce) for further immunoblotting analysis.

### Overexpression of PARP-1-DBD

The PARP-1 DBD-6 His bacterial expression vector was a gift from J.M. Pascal (Thomas Jeffeson University, Philadelphia, PA). The expression vector was inserted into the pESG-IBA3 acceptor vector (cat 3 5/4403/000, IBA, St. Louis, MO) for transfection and expression in mammalian cells. HEKn cells were transfected with the His-tagged PARP-1-DBD expression vectors using Attractene Transfection Reagent (Qiagen) according to the manufacturer's instructions. Briefly, Attractene (1 μl/ml) was added to medium containing plasmid DNA (0.12 μg/ml), incubated at room temperature for 10 min, and added dropwise to cells. Cells were incubated for 48 h to allow protein expression before treatment with arsenite. Cells transfected with PARP-1-DBD were incubated for 48 h to allow protein expression before further treatments.

### PARP-1 zinc content measurement

PARP-1 protein was isolated by immunoprecipitation and zinc content was measured with the 4-(2-pyridylazo)resorcinol (PAR) colormetric assay as previously described [8]. Briefly, after HEKn cell treatments, PARP-1 protein was purified using Dynabeads Protein A Immunoprecipitation Kit (Thermo Fisher Scientific) from cell extracts according to manufacturer's instructions. A non-denaturing method was used to elute PARP-1 from beads, and pH was adjusted to greater than 7 using the neutralizing buffer provided in the immunoprecipitation kit. Purified PARP-1 was then incubated with 10 mM hydrogen peroxide for 4 h at 4°C to release zinc. After adding 100 μM PAR, absorbance at 493 nm was recorded and used to determine relative zinc content in PARP-1 protein.

## Abbreviations

RNS: reactive nitrogen species
DSB: double strand break
PARP-1: poly(ADP-ribose) polymerase 1
iNOS: inducible nitric oxide synthase
c-PTIO: carboxy-PTIO
PAR: 4-(2-pyridylazo)resorcinol
DBD: DNA binding domain.
